# Sussex – Weight Intervention Screening for Antipsychotic Effects(WISE)

**DOI:** 10.1192/bjo.2026.11266

**Published:** 2026-06-30

**Authors:** Sabna Ahmed, Isaac Otomewo, Graham Broome

**Affiliations:** Sussex Partnership NHS Trust, Worthing, United Kingdom

## Abstract

**Aims:**

People with severe mental illness (SMI) experience disproportionate cardiometabolicmorbidity and mortality. Antipsychotic medication contributes to metabolic risk, necessitating systematic monitoring and early intervention.

Aim: was to evaluate compliance with national metabolic monitoring standards and the local WISE framework for patients prescribed antipsychotics within an inpatient rehabilitation service.

**Methods:**

A retrospective audit was conducted on 30 inpatients admitted between 16 May 2024 and 5 August 2025. Data were extracted from electronic records, laboratory systems, and a structured audit dataset. Outcomes included weight change, completion of metabolic monitoring, and documentation of interventions.

**Results:**

At admission, 76.7% were overweight/obese. Clinically significant weight gain (≥5%) occurred in 36.7% of patients. MUST screening occurred in 76.7% of cases, but only 16.7% had care plans referencing weight, and 3.3% had discharge summaries documenting weight-related plans. Metformin use was documented in 26.7% of cases with complete data.

**Conclusion:**

Weight gain during admission was common, and documentation of interventions was limited. Strengthened monitoring templates, structured care plans, and clearer escalation pathways may improve adherence to national standards and continuity of care. MUST ≠ weight-gain surveillance: it scores BMI, unintentional weight LOSS and acute disease. For antipsychotic WEIGHT GAIN, use WISE + SMI checks (baseline, weeks 4/8/12, then quarterly; annual labs) with early intervention.

